# Two Origins, Two Functions: The Discovery of Distinct Secretory Ducts Formed during the Primary and Secondary Growth in *Kielmeyera*

**DOI:** 10.3390/plants10050877

**Published:** 2021-04-27

**Authors:** Ellenhise R. Costa, Marcelo M. P. Tangerina, Marcelo J. P. Ferreira, Diego Demarco

**Affiliations:** Departamento de Botânica, Instituto de Biociências, Universidade de São Paulo, São Paulo CEP 05508-090, Brazil; ellen.costa@usp.br (E.R.C.); marcelomptang@hotmail.com (M.M.P.T.); marcelopena@ib.usp.br (M.J.P.F.)

**Keywords:** secretory ducts, structure, origin, resin, gum, evolution, *Kielmeyera*, Calophyllaceae

## Abstract

Secretory ducts have been reported for more than 50 families of vascular plants among primary and secondary tissues. A priori, all ducts of a plant are of the same type, and only slight variations in the concentration of their compounds have been reported for few species. However, two types of secretion were observed in primary and secondary tissues of *Kielmeyera appariciana*, leading us to investigate the possible influence of duct origins on the structure and metabolism of this gland. *Kielmeyera appariciana* has primary ducts in the cortex and pith and secondary ducts in the phloem. Both ducts are composed of uniseriate epithelium surrounded by a sheath and a lumen formed by a schizogenous process. Despite their similar structure and formation, the primary ducts produce resin, while the secondary ducts produce gum. This is the first report of two types of ducts in the same plant. The distinct origin of the ducts might be related to the metabolic alteration, which likely led to suppression of the biosynthetic pathway of terpenoids and phenolics in the secondary ducts. The functional and evolutionary implications of this innovation are discussed in our study and may be related to the diversification of *Kielmeyera* and Calophyllaceae in tropical environments.

## 1. Introduction

Secretory ducts are internal glands composed of an epithelium formed by secretory cells that release the exudate in an elongated intercellular space called lumen [1]. They occur in 54 families of vascular plants, with a prevalence of resin ducts in 48 families [2,3] and six families with mucilage ducts: Welwitschiaceae, Chloranthaceae, Combretaceae, Malvaceae, Neuradaceae, and Vochysiaceae [4,5,6,7,8]. Secretory ducts are particularly common in families, such as Anacardiaceae, Asteraceae, Burseraceae, Calophyllaceae, Clusiaceae, and Salicaceae, and some Fabaceae and Malvaceae [1,3,9,10,11,12,13,14].

Ducts may originate from ground meristem, procambium and/or cambium [1] and vary from slightly elongated structures, as found in some Asteraceae, Malvaceae, and Salicaceae [12,13,15], to extremely long, continuous ducts throughout the entire plant, as observed in conifers, Anacardiaceae, and Burseraceae [13]. Nevertheless, distinct secretory ducts within an organ in each species produce the same type of secretion in almost all plants, regardless of their origin [1,3,16].

While the secretion of the ducts has a variable composition in the species, they can be generically grouped into three types: resin, mucilage, and gum [1,3,10], and each type of secretion is usually conservative within the families [3,9], often being used as a diagnostic character [17]. Few families have distinct genera producing different secretions in their respective ducts, such as Anacardiaceae with resin ducts in almost all genera and gum ducts in *Lannea*, *Operculicarya,* and *Rhodosphaera* [18,19]. Considering the resin ducts, only small variations in the secretion composition have been observed comparing ducts of vegetative and reproductive organs in the same species, inferred from a different colour of the exudate in each organ or based on the ultrastructure of the epithelial cells [20,21]. Different types of ducts occurring side by side have never been reported for Malpighiales until now. However, our field observations remarkably showed two very distinct types of secretion being exuded by leaves and stems in secondary growth in *Kielmeyera appariciana* Saddi.

*Kielmeyera* is one of the largest genera of Calophyllaceae, comprising 50 species, the leaf and bark extracts of which are used in folk medicine as anti-inflammatory, antioxidant, antibacterial, and antifungal agents. Recently, biological assays have confirmed the efficacy of *Kielmeyera* extracts even against carcinogenic cell strains [22,23,24]. The main secretion found in the genus is resin, which is produced by secretory ducts located in all organs, mainly adjacent to the vascular system [9].

Secretory ducts occur in all species of Calophyllaceae, and there are divergences with respect to their secretion, previously referred to as resin, gum, or latex [9,25,26,27]. These divergences raise doubts about the type of duct present in the family and about its possible diversity. Additionally, there are still many doubts in the interpretation of the anatomy of these secretory structures as well as the chemical nature of the secreted compounds.

Therefore, we selected *K. appariciana* as a model to investigate the structure of its ducts occurring in primary shoots and stems in secondary growth, the chemical nature of their exudate, as well as the origin of these ducts and the possible influence of their origin on their metabolism.

## 2. Results

**Figure 1:**
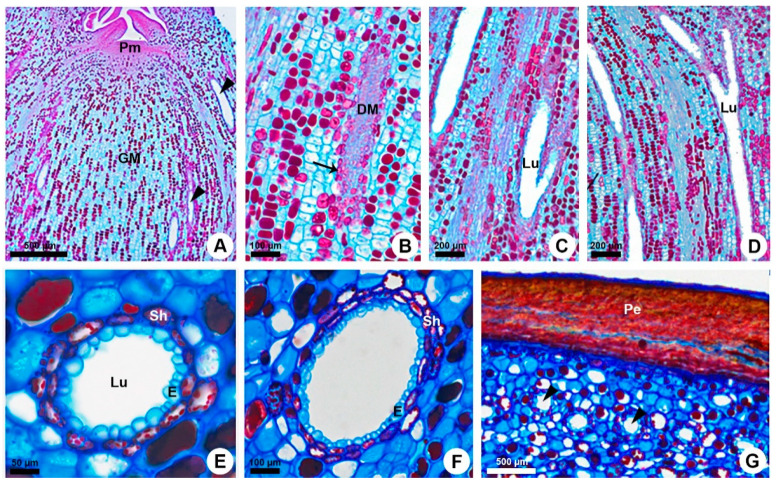
Origin, distribution, and structure of the primary resin ducts in *Kielmeyera appariciana*. Astra blue and safranin staining. (**A**–**D**) Longitudinal sections. (**E**–**G**) Transverse section. (**A**) Origin of the primary resin ducts in cortex and pith of the shoot apex. (**B**) Strand of meristematic cells derived from ground meristem, which will form the duct lined by a phenolic sheath (arrow). (**C**,**D**) Resin ducts in the parenchyma. Note the branched ducts formed by lateral fusion of two adjacent ducts. (**E**,**F**) Primary resin ducts surrounded by a phenolic sheath in the cortex (**E**) and pith (**F**). (**G**) Narrow resin ducts in the outer cortex. Arrowhead = resin duct; DM = duct meristematic cells; E = epithelium; GM = ground meristem; Lu = lumen; Pe = periderm; Pm = promeristem; Sh = duct phenolic sheath.

**Figure 2:**
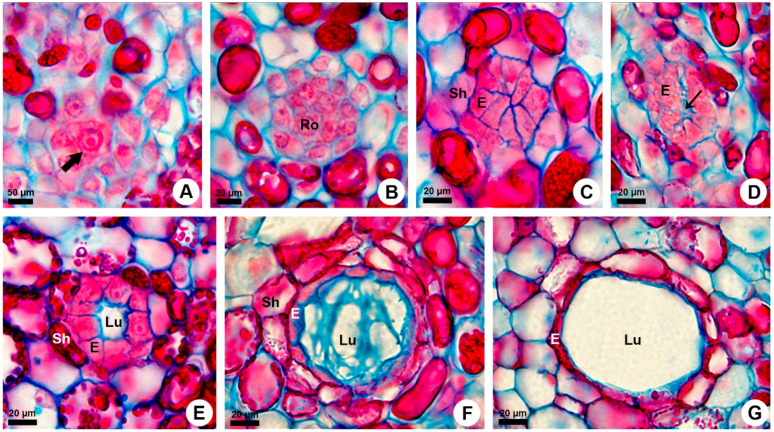
Development of the primary resin ducts in *Kielmeyera appariciana*. Astra blue and safranin staining. Transverse sections of shoot apex. (**A**–**G**) Stages of formation of the primary resin ducts. (**A**) Initial cell (wide arrow). (**B**) Rosette formation. (**C**) Differentiation of epithelium and sheath. (**D**) Initial formation of the lumen (narrow arrow). (**E**) Beginning of the lumen expansion. (**F**) Young duct filled with secretion. (**G**) Mature duct. Note the flattened epithelial cells. (E = epithelium; Lu = lumen; Ro = rosette; Sh = duct sheath).

**Figure 3:**
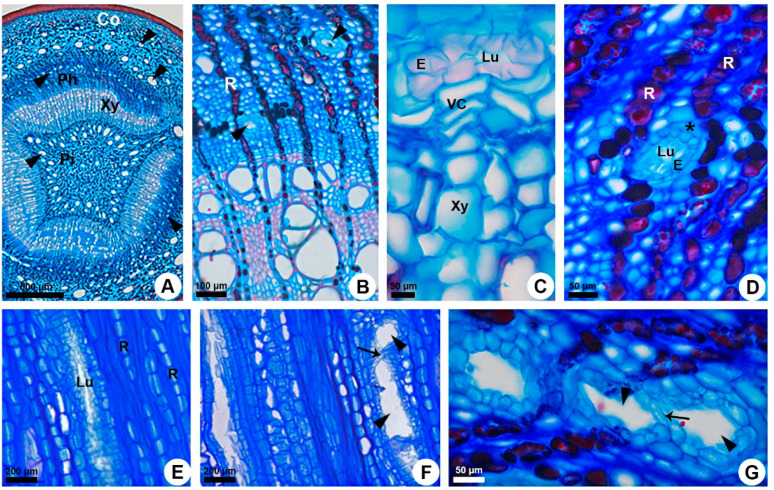
Origin, distribution, and structure of the secondary gum ducts in *Kielmeyera appariciana*. Astra blue and safranin staining. (**A**–**D**,**G**) Transverse sections. (**E**,**F**) Tangential longitudinal sections. (**A**) Overview of the stem in secondary growth. (**B**) Gum ducts in the secondary phloem. (**C**) Origin of the secondary gum ducts from vascular cambium. (**D**) Detail of the duct filled with secretion, surrounded by a parenchyma sheath. Note the displacement of the uniseriate rays due to duct expansion. (**E**) Gum duct in the axial parenchyma of secondary phloem. (**F**,**G**) Branched ducts. Ducts branch by lateral anastomose of the contact cells (narrow arrow) of two adjacent ducts. Arrowhead = duct; asterisk = parenchyma sheath; Co = cortex; E = epithelium; Lu = lumen; Ph = phloem; Pi = pith; R = ray; VC = vascular cambium; Xy = xylem.

*Kielmeyera appariciana* has primary and secondary ducts throughout the shoot system (Figure 1, Figure 2 and Figure 3), which form an extensive secretory network that protects all aerial parts of the plant against herbivory.

### 2.1. Primary Ducts

The primary ducts are widely distributed in the cortex and pith (Figure 1A,B). They are axially elongated and vary in diameter and length (Figure 1C–G). The ducts occurring in the outer cortex are very narrow (Figure 1G) compared to the ducts located closer to the phloem (Figure 1E). On the other hand, the medullary ducts are always wide (Figure 1F) and mainly distributed close to the vascular system (Figure 1A). Several ducts merge laterally, and some of them even bifurcate, especially in the nodes, where they are continuous between stem and leaf. These merged ducts may or may not split further (Figure 1C,D).

Each duct is composed of uniseriate secretory epithelium comprised of thin-walled cells with dense cytoplasm and prominent nucleus (Figure 2E,F). Additionally, the duct has a sheath that varies from uni- to biseriate and contains phenolic compounds (Figure 1E,F or Figure 2E,F).

Ontogenetic analyses of the shoot apices of *K. appariciana* revealed that the primary ducts are formed just below the shoot apical meristem after the differentiation of the procambium in the cortical region (Figure 1A). The medullary ducts are formed soon after the origin of the cortical ducts. Primary ducts originate from a single cell of the ground meristem that has thin walls, dense cytoplasm, and prominent nucleus (Figure 2A). Successive divisions of this initial cell form a rosette of undifferentiated cells that remain in constant division (Figure 2B). The rosette cells are actually arranged in an elongated strand (Figure 1B), which starts to differentiate into two distinct regions. The central cells will compose the duct epithelium, while the peripheral cells will give rise to the phenolic sheath (Figure 2C).

During the differentiation of the epithelium, a small aperture in the central region of the rosette is formed by separation of cells (Figure 2D). Later, this aperture expands schizogenously, giving rise to the lumen of the duct (Figure 2E). Concomitantly, epithelial cells begin to produce secretion, which is released into the expanding lumen (Figure 2F). At this secretory phase, the epithelial cells are slightly elongated inwards, sinuously outlined (Figure 2D–F), with cytoplasm filled with secretion. At the final stage of development, mature ducts stop producing secretion, and the epithelium flattens (Figure 2G).

### 2.2. Secondary Ducts

Secondary ducts occur in the secondary phloem (Figure 3A–G). They are narrower than the primary ones (Figure 3A), and larger diameters are only observed when two or more adjacent ducts merge laterally (Figure 3F,G). These ducts are located in axial parenchyma bands with a stratified arrangement (Figure 3A,B). Structurally, secondary ducts are similar to the primary ones, with uniseriate epithelium surrounded by a sheath (Figure 3B–D) but, in this case, the sheath is parenchymatic (Figure 3D).

Unlike the primary ducts, the secondary ducts are formed by a set of cells. This set is composed of fusiform initials of the vascular cambium that differentiate very early and form the duct lumen by schizogeny (Figure 3C). Some ducts merge giving rise to branched ducts (Figure 3F,G), always within the axial parenchyma. There is no radial duct in *K. appariciana* (Figure 3E), and when the ducts are formed in the direction of the rays, it is observed that the expansion of the lumen affects the path of the ray, which becomes sinuous at this point (Figure 3B,D) and reaches the duct (Figure 3D).

### 2.3. Secretion Composition

**Figure 4:**
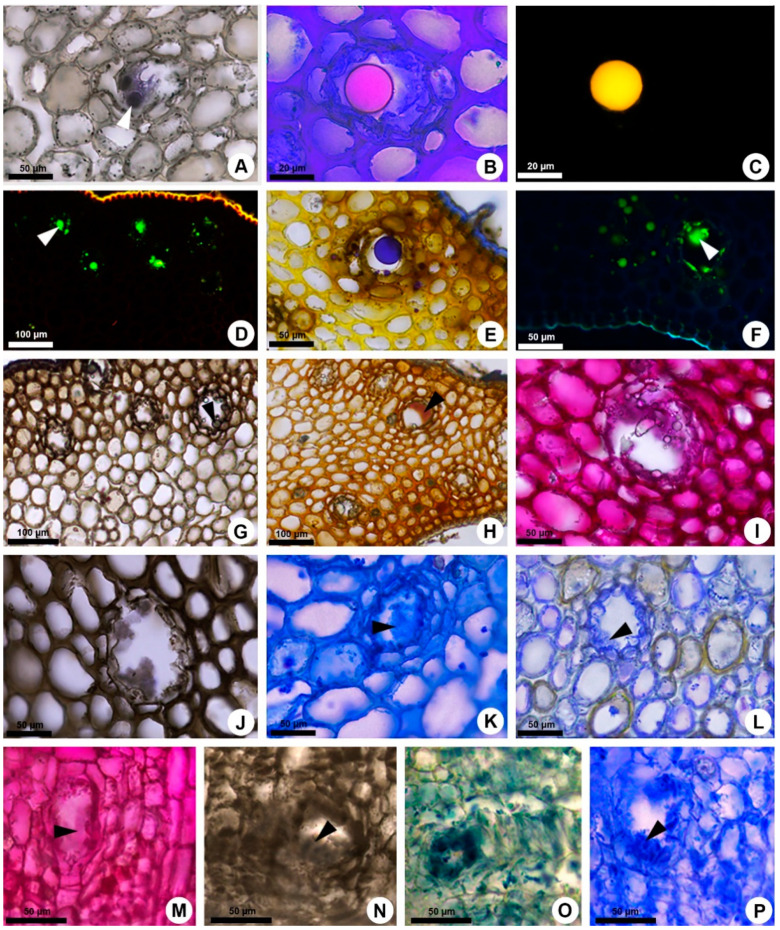
Histochemical analysis of the secretion of primary and secondary ducts of *Kielmeyera appariciana*. (**A**–**L**) Primary resin ducts. (**M**–**P**) Secondary gum ducts. (**A**) Lipids stained with Sudan black. (**B**,**C**) Neutral lipids identified by Nile blue in bright field (**B**) and under blue light (**C**). (**D**) Lipids detected by neutral red under blue light. (**E**) Essential oils identified by NADI reagent. (**F**) Autofluorescence of the secretion under UV. (**G**,**H**) Phenolic compounds detected using ferric chloride (**G**) and potassium dichromate (**H**). (**I**–**K**,**M**,**N**) Polysaccharides identified using ruthenium red (**I**,**M**), tannic acid and ferric chloride (**J**,**N**) and Alcian blue (**K**). (**L**,**O**,**P**) Proteins detected by coomassie blue (**L**,**P**) and aniline blue black (**O**). Arrowhead = secretion within the duct.

The differences between the primary and secondary ducts of *K. appariciana* are not restricted to the origin. Field observations showed that the secretion exuded from both ducts is initially translucent and viscous; however, shortly after exposure of this exudate to air, a polymerization of the secretion is observed. The exudation of primary ducts in developing leaves and stems tends to solidify and harden, while the secretion exuded by secondary ducts in stems in secondary growth polymerizes and acquires a gelatinous consistency. Histochemically, the secretions are very distinct. Primary ducts produce resin composed of lipids (Figure 4A–F), including essential oils (Figure 4E), phenolic compounds (Figure 4G,H), polysaccharides (Figure 4I–K) and proteins (Figure 4L), while secondary ducts secrete only gum comprised of polysaccharides (Figure 4M,N) and proteins (Figure 4O,P).

### 2.4. Chemical Analysis

**Figure 5:**
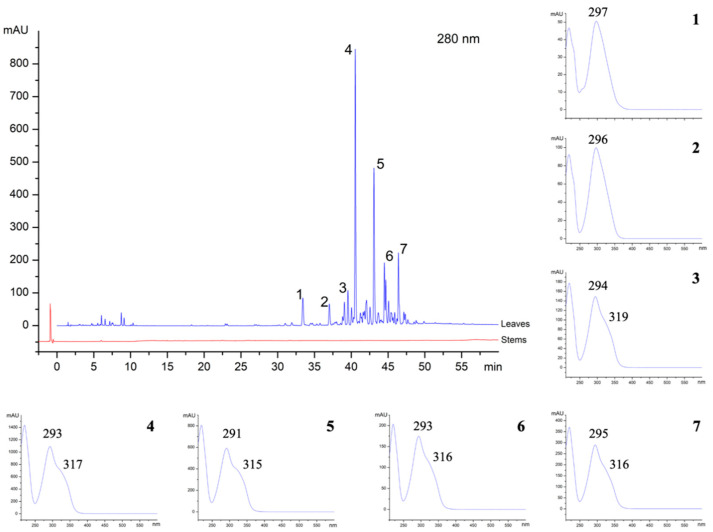
HPLC-DAD analyses of exudate secretion of *Kielmeyera appariciana* leaves and stems and observed UV spectra of major peaks.

Chemical analysis also confirmed the different composition of the secretion from ducts with distinct origin. Both secretions were analysed through HPLC-DAD. From the overlapping of chromatograms obtained from leaves and stems in secondary growth exudates of *K. appariciana*, it is possible to verify the presence of various phenolic compounds only in the leaf exudate (Figure 5). Even when injecting the sample from stems in secondary growth in a higher concentration, these compounds were not detected. The phenolic compounds were revealed by their characteristic UV spectrum [28], and the main peaks observed in the chromatogram showed a very similar UV spectrum (Figure 5). Through HPLC-MS analysis, the seven major peaks observed showed the following *m/z* values: (1) Retention time (R_t_): 33.41 min., *m/z* 359.1497; (2) R_t_: 37.01 min., *m/z* 373.1653; (3) R_t_: 39.05 min., *m/z* 387.1809; (4) R_t_: 40.55 min., *m/z* 359.1502; (5) R_t_: 43.08 min., *m/z* 343.1560; (6) R_t_: 44.71 min., *m/z* 373.1656; (7) R_t_: 46.44 min., *m/z* 357.1710, according to mass spectra shown in the Supplementary Material (Figures S1–S7). A spectral library search available at the GNPS website (Global Natural Products Social Network: gnps.ucsd.edu) [29] did not indicate a correspondence with any known compound.

## 3. Discussion

Our study demonstrated for the first time the existence of two types of secretory ducts within a plant. *Kielmeyera appariciana* has resin ducts in the primary stem and leaves and gum ducts in the secondary phloem of the stem.

In the majority of plants with secretory ducts, these ducts are located in the primary and secondary regions of the plant body since most ducts occur in the vascular system [9]. In fact, in 40 of the 54 families with secretory ducts, these ducts are found in primary and secondary vascular systems [9]. Ducts occur in five families of Malpighiales—Calophyllaceae, Clusiaceae, Hypericaceae, Humiriaceae, and Salicaceae. All five families have fundamental primary ducts in the cortex and pith but Calophyllaceae, Clusiaceae, and Hypericaceae may also have vascular ducts in the secondary phloem. There are rare reports of the presence of ducts in the primary phloem of some Clusiaceae and in the wood rays of *Mammea* (Calophyllaceae) and *Garcinia* (Clusiaceae) [2,3,9]. The occurrence of secretory structures only in primary tissues is common for some types of glands [1], but the occurrence of one type of secretory structure only in secondary tissues is extremely rare and has only been reported for laticifers of Hippocastanoideae (Sapindaceae) [30]. The other only report is the traumatic resin ducts of the wood of some conifers. However, in this case, the ducts are only formed under injury [1]. The occurrence of glands in the secondary vascular system (i.e., originated by cambium) is expected when the same type of gland also occurs in the primary vascular system (i.e., originated by procambium). Thus, the observation of fundamental primary ducts followed by secondary phloem ducts, as noted in *Kielmeyera*, is not common. Few genera have this type of duct distribution in different tissue systems when comparing primary and secondary regions of the plant body, as observed in *Pinus*, which has primary ducts in the cortex and secondary ducts in the xylem [31].

### 3.1. Distribution within the Plant

The ducts of *K. appariciana* form a network across the entire shoot system in the cortex, pith, and secondary phloem. This wide distribution constitutes an efficient defensive system against herbivory since any region of the plant that is injured will cause the exudation of the internal secretion. A similar distribution of the primary ducts along the axial system of the stem has also been reported for *Parthenium argentatum* [32], *Commiphora wightii* (Arn.) Bhandari [33], and *Lannea coromandelica* (Houtt.) Merr. [18]. These ducts may have varied arrangements, such as vertical, horizontal, or irregular orientation, and be continuous or discontinuous, branched, or unbranched, according to Venkaiah and Shah [18]. Ducts of *K. appariciana* form a system of continuous branched tubes, which fuse apically and laterally, contributing to a significant expansion of the duct in length and width.

Secondary ducts are located in axial parenchyma and have also been referred to as vertical ducts by some authors, such as Wu and Hu [31] and Sato and Ishida [34]. These ducts occur within the axial parenchyma bands of the secondary phloem, which may be related to growth layers, as occurs in the wood of *Copaifera langsdorffii* [35]. Radial ducts are common in some families, such as Pinaceae and Anacardiaceae [14,31], but they are absent in *Kielmeyera*, the ray of which is displaced when a duct is formed in its direction. The occurrence of ducts that are closely linked to the parenchyma rays is common. Wiedenhoeft and Miller [36] identified the same relation between ducts and rays and warned that even if ray cells pass very close to both sides of the duct, they should not be considered part of the duct.

### 3.2. Duct Diversity

Histologically, the ducts are very similar to each other, and their diversity is related to their mode of formation of the lumen or to the type of secretion produced. The mode of formation may be of three types: schizogenous, lysigenous, or schizo-lysigenous [1,37,38,39]. The schizogenous mode occurs when the lumen is formed exclusively by cell separation, as observed in the primary and secondary ducts of *K. appariciana*. On the other hand, the lysigenous mode occurs when the lumen is formed by programmed cell death of one or more cells of the rosette. Finally, the schizo-lysigenous mode occurs when both processes occur for the formation of the lumen [1]. The formation mode of ducts varies from species to species [40] and may also vary in different regions of the same organ [18]. However, even when the same secretory structure has different origins in a plant, the nature of secretion is very similar, as reported by several authors [1,20,21,41,42,43,44]. The only exception was recently reported for secretory ducts in two species of Anacardiaceae [14].

In relation to the diversity of exudates, ducts can produce resin, mucilage, or gum [1,3,10,13,14], with a great diversity of composition for the secretions classified as resin (broad sense) [14], which is always mostly lipophilic (terpenic or rarely phenolic) [3]. The wide distribution of resin ducts in vascular plants is directly related to the type of environment in which the groups of resinous plants have evolved, such as tropical environments where the rate of herbivory is higher [3,10], and may explain the chemical diversity found in some groups.

### 3.3. Origin and Metabolism

Our results showed that the differences between the primary and secondary ducts of *K. appariciana* are not restricted only to their origin from ground meristem or cambium. Resin is only produced in cortex and pith, which is mainly composed of terpenes and phenolics but also contains polysaccharides and proteins. Conversely, gum is produced in secondary phloem, where the production of lipophilic compounds has likely been suppressed. Our chemical analysis confirmed the different composition of the secretions produced by each duct.

Some factors may be involved in this unusual metabolic alteration. Our hypothesis is the suppression of some genes related to production of terpenoids, such as the terpenoid synthase (TPS) genes, and phenolics, such as phenylalanine ammonia lyase (PAL) genes. TPS is a superfamily of genes conserved in gymnosperms and angiosperms and is likely derived from a single ancestor [45,46]. Accordingly, phenolic acids in plants are primarily derived from the shikimate biosynthetic pathway with the conversion of phenylalanine to cinnamic acid by phenylalanine ammonia lyase (PAL) [47]. Changes in TPS and PAL gene sequences or in their gene expression may be related to the origin of two types of ducts in *K. appariciana*, which could be an interesting initial hypothesis to be investigated in future studies.

The regulation of plant terpenoid biosynthesis is generally related to spatial and temporal aspects, and developmental regulation has already been reported in the production of some terpenoids [46,48,49,50]. In addition, changes in gene regulation that alter terpene quantities are linked with functional shifts, according to Theis and Lerdau [51], and might have conferred adaptive advantages to *Kielmeyera*.

### 3.4. Function

Functionally, the occurrence of two types of secretory ducts in the same plant may represent a specialization of the secretory system of the plant in relation to its ontogenetic stage. While primary resin ducts protect leaves and stem against herbivores during early development of the shoot system, secondary gum ducts containing plentiful polysaccharides assist in the retention and/or translocation of water from the xylem into the phloem [1,52,53]. In general, the marked combination of phenolic compounds and polysaccharides in different regions of the plant provides advantages for the plant as a whole because of its ability to absorb and economize water and protect against herbivory [54]. The presence of phenolics in primary shoots indicates that the species invests in the protection of its photosynthetic organs against herbivore attacks since the most predated plant organ is usually the leaf [55].

### 3.5. Evolutionary Implications

**Figure 6:**
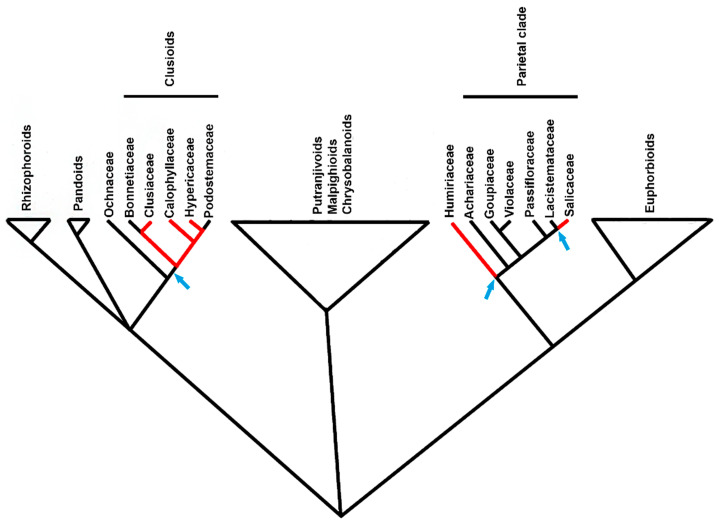
Simplified cladogram of Malpighiales, adapted from Xi et al. [57], showing the multiple emergences of the secretory ducts in the order (arrows). Red lines represent the presence of secretory ducts and black lines indicate their absence.

The evolutionary emergence of two types of ducts in *K. appariciana* represents an apomorphic character of *Kielmeyera*, which may be related to genus diversification and should be researched in other species to evaluate its occurrence in the clade. Secretory ducts have evolved at least three times independently in Malpighiales, occurring in Calophyllaceae, Clusiaceae, Humiriaceae, Hypericaceae, and Salicaceae. Secretory ducts have evolved once in the clusioid clade with two reversals in Bonnetiaceae and Podostemaceae and two other emergences in Humiriaceae and Salicaceae in the parietal clade (Figure 6). The formation mode of these ducts is quite distinct in each clade. In the clusioid clade, as observed in *Kielmeyera*, ducts are formed by a strand of meristematic cells, identified as a rosette in transverse sections, as described for most families, but ducts of Humiriaceae (pers. obs.) and Salicaceae [56] are formed by coalescence of cavities originating various transitional shapes between cavities and ducts, as recently described for Malvaceae [13].

Despite this being the first investigation on two types of ducts distinguished by origin and secretory metabolism in the same plant, further studies are needed and lead us to new questions about the relation between the origin and the secretory activity in plant glands, especially in secretory ducts.

## 4. Materials and Methods

### 4.1. Plant Material

Samples of *Kielmeyera appariciana* Saddi were collected from the campus of the Universidade de São Paulo in São Paulo/SP (Brazil) and the voucher was deposited in the Herbarium SPF (Costa, E.R. 1).

### 4.2. Histological Analysis

Several primary shoots with leaves at different developmental stages (leaf primordium, developing leaves, and mature leaves) and fragments of stems in secondary growth (more than 1 cm of diameter) were collected and fixed in Karnovsky’s solution for 24 h at 4 °C for the structural analyses. After fixation, shoot apices and stem portions in secondary growth were isolated, dehydrated through a tertiary butyl alcohol series [58], embedded in Paraplast (Leica Microsystems Inc., Heidelberg, Germany), and serial sectioned at 10 µm thickness on a Leica RM2145 rotary microtome. Longitudinal and transverse sections were stained with astra blue and safranin O [59] and the slides were mounted with Permount (Fisher Scientific, Pittsburgh, PA, USA).

Fresh shoot apices and stems in secondary growth were also free-hand sectioned for histochemical analyses of the secretion. The following histochemical tests were applied: Sudan black B and Sudan IV [60] in bright field and neutral red under blue light [61] for lipids, Nile blue [62] in bright field and under blue light for neutral and acidic lipids, Nadi reagent [63] for terpenoids (essential oils and resin), copper acetate and rubeanic acid [64,65] for fatty acids, ferric chloride [58] and potassium dichromate [66] for phenolic compounds, vanillin and hydrochloric acid [67,68] for tannins, Dragendorff’s reagent [69] and Wagner’s reagent [70] for alkaloids; periodic acid—Schiff reaction (PAS) [71] for polysaccharides, ruthenium red [72] and Alcian blue [60] for acidic mucilage, tannic acid and ferric chloride [73] for mucilage, and coomassie blue and aniline blue black [74] for proteins. The autofluorescence of the secretion was also analysed under UV and blue light. All tests and their respective control procedures were carried out according to Demarco [75].

Observations and photographs were performed using a Leica DMLB light microscope equipped with an HBO 100W mercury vapor lamp and a blue light filter block (excitation filter BP 420–490, dichromatic mirror RKP 510, suppression filter LP 515) and UV filter block (excitation filter BP340-380, dichromatic mirror RKP400, suppression filter LP425).

### 4.3. Chemical Analysis

For chemical analysis, duct exudates from mature leaves and from stems in secondary growth (more than 3 cm of diameter) were collected directly from the plant. Thus, the leaves and stems were cut using a razor and drops of the exudates were collected in separate beakers. Samples were diluted in distilled water and filtered through a C_18_ cartridge, first eluted with 9 mL of H_2_O followed by 9 mL of methanol. Fractions were dried and analysed by high performance liquid chromatography coupled to photodiode array detector (HPLC-DAD) using solvents A (H_2_O + 0.1% acetic acid) and B (acetonitrile HPLC grade, J. T. Baker^®^). Analyses were performed on an Agilent 1260 chromatograph (Agilent Technologies Inc., Santa Clara, CA, USA) equipped with a 60 mm flow cell and a Zorbax Eclipse plus reverse phase C_18_ (4.6 × 150 mm) column containing 3.5 μm particle diameter as the stationary phase. All analyses were carried out on a mobile phase flow rate of 1.0 mL·min^−1^, 45 °C of temperature, and 3 μL of sample injection at 2 mg·mL^−1^ of concentration. The chromatographic run method used consisted of: 10–25% B in 10 min, followed by 25–50% B in 20 min, and 50–100% B in 20 min, maintaining 100% B for an additional 10 min, in a total of 60 min. HPLC-MS (high performance liquid chromatography coupled to mass spectrometry) analyses were performed on a Shimadzu chromatograph (Shimadzu Corporation, Kyoto, Japan) coupled to a MAXIS 3G—Bruker Daltonics Q-TOF mass spectrometer (Bruker Corporation, Billerica, MA, USA) with capillary voltage of 4500 V and nebulizer at 27 psi in negative mode. The same chromatographic method previously described was applied, allowing to assign the *m/z* values of the peaks observed in the chromatogram obtained by HPLC-DAD. Data from HR-MS analysis are shown in the Supplementary Material (Figures S1–S7).

## 5. Conclusions

Our study demonstrated for the first time in Malpighiales the existence of two types of secretory ducts within a plant. *Kielmeyera appariciana* has resin ducts in primary organs but start producing gum ducts during secondary growth. The distinct origin of the secondary ducts might be related to suppression of the secretory pathway of terpenoids and phenolics in the epithelial cells, resulting in two types of ducts, even though their similar structure and formation mode. The more diverse defence secretory system of *Kielmeyera* may be associated with genus diversification. These results reinforce the importance of developmental studies and raise new questions about the possible relationship between origin and metabolism in plant glands.

## Data Availability

All figures and tables of this manuscript have been unpublished and were made specifically for this article.

## Supplementary Materials

The following are available online at https://www.mdpi.com/article/10.3390/plants10050877/s1; Figures S1–S7: High-resolution mass spectra obtained in negative mode of compounds corresponding to peaks 1 to 7, respectively.
