# Supplementary material for: Two Origins, Two Functions: The Discovery of Distinct Secretory Ducts Formed during the Primary and Secondary Growth in Kielmeyera
Source: Plants (Basel). 2021 Apr 27;10(5):877. doi: 10.3390/plants10050877 (PMC8146764; doi:10.3390/plants10050877)
Supplement: Supplementary file 1 [file plants-10-00877-s001.zip › plants-1185426-supplementary.pdf]

Supplementary Material

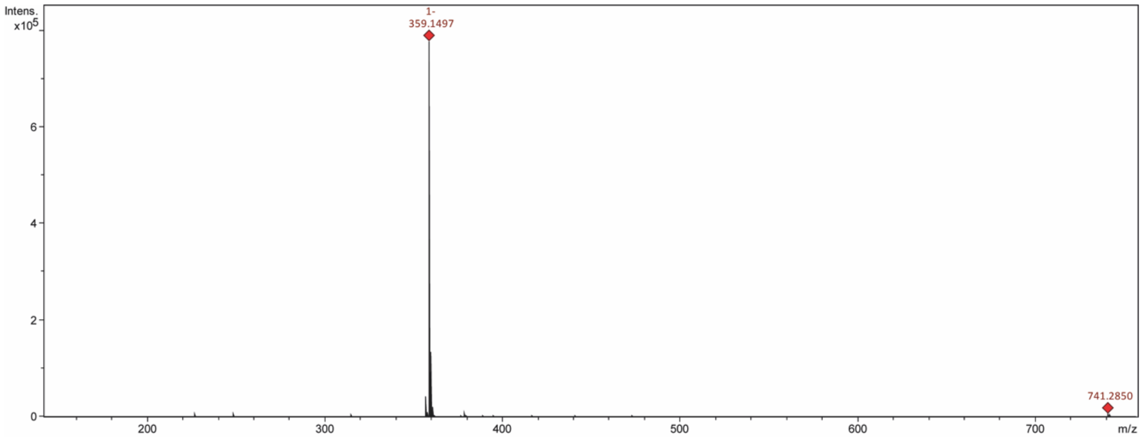

Figure S1. High-resolution mass spectrum of compound corresponding to peak 1, negative mode.

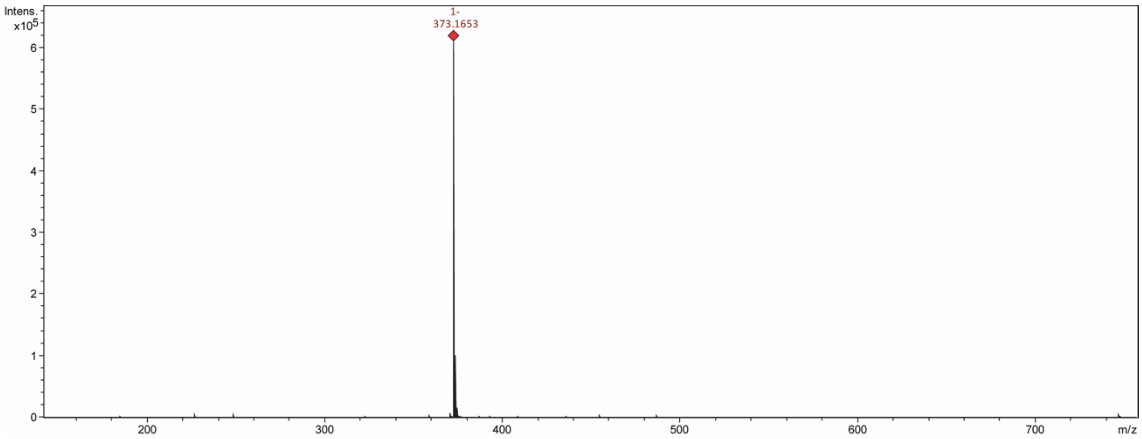

Figure S2. High-resolution mass spectrum of compound corresponding to peak 2, negative mode.

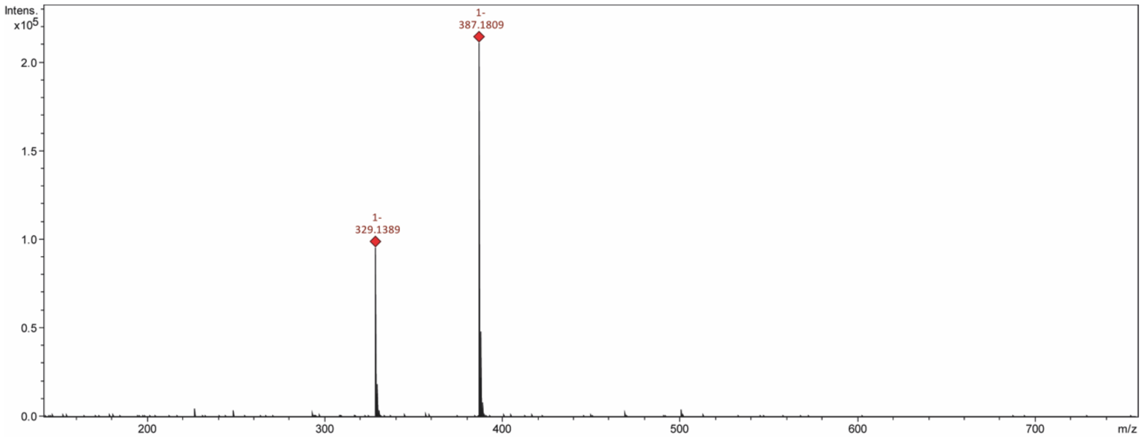

Figure S3. High-resolution mass spectrum of compound corresponding to peak 3, negative mode.

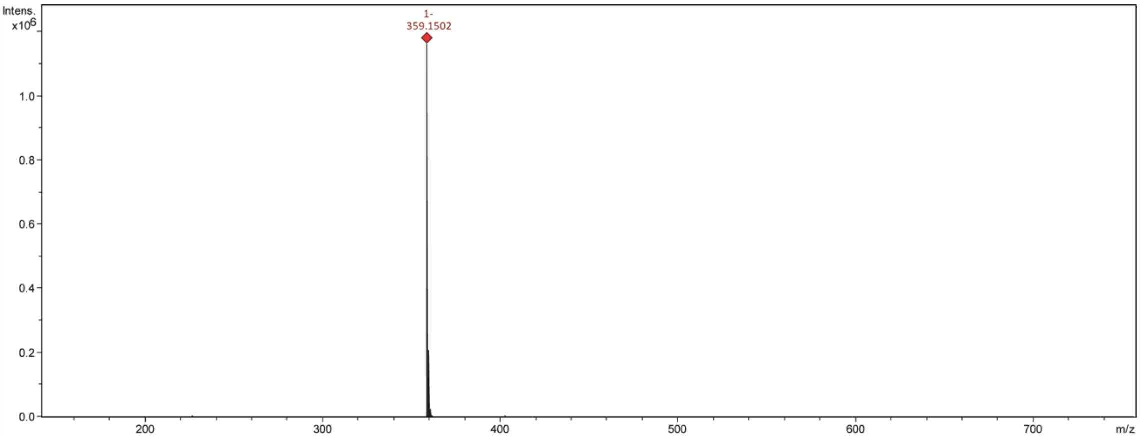

Figure S4. High-resolution mass spectrum of compound corresponding to peak 4, negative mode.

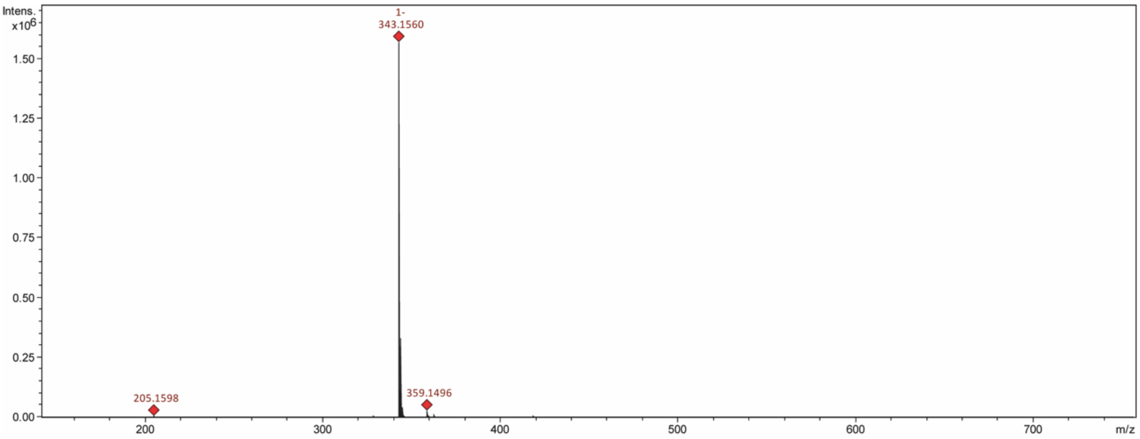

Figure S5. High-resolution mass spectrum of compound corresponding to peak 5, negative mode.

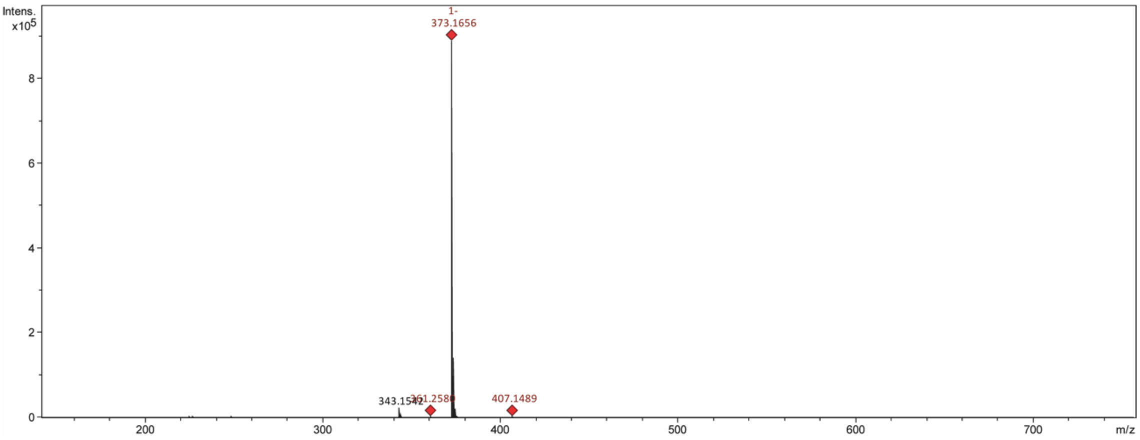

Figure S6. High-resolution mass spectrum of compound corresponding to peak 6, negative mode.

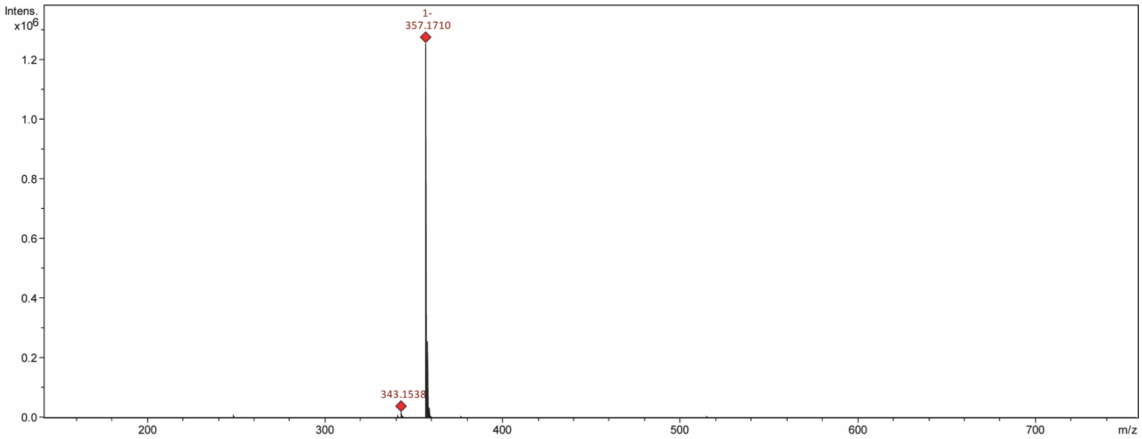

Figure S7. High-resolution mass spectrum of compound corresponding to peak 7, negative mode.
